# Comparative analysis of differentially expressed genes in normal and white spot syndrome virus infected *Penaeus monodon*

**DOI:** 10.1186/1471-2164-8-120

**Published:** 2007-05-16

**Authors:** Jiann-Horng Leu, Chih-Chin Chang, Jin-Lu Wu, Chun-Wei Hsu, Ikuo Hirono, Takashi Aoki, Hsueh-Fen Juan, Chu-Fang Lo, Guang-Hsiung Kou, Hsuan-Cheng Huang

**Affiliations:** 1 Institute of Zoology, National Taiwan University, Taipei 106, Taiwan; 2 Institute of Bioinformatics, National Yang-Ming University, Taipei 112, Taiwan; 3 Graduate School of Marine Science and Technology, Tokyo University of Marine Science and Technology, 4-5-7 Konan, Minato, Tokyo 108-8477, Japan; 4 Department of Life Science, Institute of Molecular and Cellular Biology, National Taiwan University, Taipei 106, Taiwan; 5 Department of Biological Sciences, National University of Singapore, 117543, Singapore

## Abstract

**Background:**

White spot syndrome (WSS) is a viral disease that affects most of the commercially important shrimps and causes serious economic losses to the shrimp farming industry worldwide. However, little information is available in terms of the molecular mechanisms of the host-virus interaction. In this study, we used an expressed sequence tag (EST) approach to observe global gene expression changes in white spot syndrome virus (WSSV)-infected postlarvae of *Penaeus monodon*.

**Results:**

Sequencing of the complementary DNA clones of two libraries constructed from normal and WSSV-infected postlarvae produced a total of 15,981 high-quality ESTs. Of these ESTs, 46% were successfully matched against annotated genes in National Center of Biotechnology Information (NCBI) non-redundant (nr) database and 44% were functionally classified using the Gene Ontology (GO) scheme. Comparative EST analyses suggested that, in postlarval shrimp, WSSV infection strongly modulates the gene expression patterns in several organs or tissues, including the hepatopancreas, muscle, eyestalk and cuticle. Our data suggest that several basic cellular metabolic processes are likely to be affected, including oxidative phosphorylation, protein synthesis, the glycolytic pathway, and calcium ion balance. A group of immune-related chitin-binding protein genes is also likely to be strongly up regulated after WSSV infection. A database containing all the sequence data and analysis results is accessible at .

**Conclusion:**

This study suggests that WSSV infection modulates expression of various kinds of genes. The predicted gene expression pattern changes not only reflect the possible responses of shrimp to the virus infection but also suggest how WSSV subverts cellular functions for virus multiplication. In addition, the ESTs reported in this study provide a rich source for identification of novel genes in shrimp.

## Background

White spot syndrome (WSS) is a highly contagious viral disease of penaeid shrimp. The cumulative mortality of diseased shrimp can reach 100% within 3–10 days. Since its first outbreak in 1993, WSS has caused serious economic losses to the shrimp farming industry worldwide. The causative agent, white spot syndrome virus (WSSV), is an enveloped, non-occluded, rod-shaped virus that contains a circular, double-stranded DNA of about 300 kb. This virus has an extremely wide range of potential hosts, infecting not only shrimps, but also other decapods [1,2]. WSSV infects most shrimp tissues and organs, and it replicates in the nuclei of infected cells. At the late stage of infection, either the nucleus or the whole cell disintegrates, leading to loss of cellular architecture. Both genomic and proteomic approaches have revealed the unique characteristics of the virus, and the virus has been erected as the type species of the new family of *Nimaviridae *[3-5].

Due to its serious impact on shrimp aquaculture, there is an urgent need to understand WSSV and to unveil the underlying mechanisms involved in WSSV pathogenesis in shrimp. Although considerable progress has been made in characterizing the virus, information on the host genes involved in WSSV pathogenesis is limited. To identify these host genes, one strategy is to isolate genes that are differentially expressed after WSSV infection. To that purpose, a variety of different approaches have been used, including an mRNA differential display technique [6], suppression subtractive hybridization [7], SSH and differential hybridization [8], cDNA microarrays [9,10] and ESTs [11]. Both cDNA microarrays and EST libraries are particularly suitable for large-scale gene expression analysis, and both of these methods have been well developed in several model organisms. However, the application of these methods to shrimp is still in its infancy. Consequently, compared to other model organisms, only relatively few sequenced ESTs and microarray cDNA targets are available for shrimp. Furthermore, all of the studies cited above focused exclusively on the identification of immune-related genes with only immune-related organs, (ie, the hemocytes and the hepatopancreas [HP]) being analyzed. However, WSSV is a systemic virus that infects most shrimp tissues and organs, and it is logical to assume that in different cell types, the virus would be likely to modulate the expression of different host genes in order to promote its multiplication in the correspondingly different cellular contexts. If so, then the gene expression changes induced by WSSV in immune-related cells should be different from those in non-immune cells. Therefore, in the present paper, rather than using a specific tissue or organ to investigate only the gene expression patterns of immune-related cells, we instead take a global view by using entire *P. monodon *postlarvae as our study subject. Our large scale EST approach used two different cDNA libraries, one from normal and one from WSSV-infected *P. monodon *postlarvae. The respective EST data were then compared to predict the gene expression changes in host shrimp after WSSV infection.

As an additional benefit, this large scale EST study also increases our transcriptomic data for Crustacea and penaeid shrimp. This in turn will improve our understanding of penaeid shrimp biology, which is important because the penaeid shrimp are economically valuable and yet they remain vulnerable to outbreaks of various viral diseases. Research into the genetics and genomics of shrimp has been gaining in importance over the past decade, and a number of penaeid shrimp EST projects have already been undertaken. However, most of these projects and their associated EST libraries were small in scale [12-15]. Currently, the two largest penaeid shrimp EST studies have published 13,656 and 10,100 ESTs, respectively [16,17]. We hope that, together with the 15,981 additional ESTs released with this report, this will provide a good foundation for further research into the genetics, genomics and even the proteomics of shrimp.

## Results

### Generation and analysis of EST libraries

Two cDNA libraries, PmTwN and PmTwI, were constructed from normal and WSSV-infected postlarvae of *P. monodon*, respectively. No normalization was applied to these two libraries. A total of 7,200 and 8,064 clones were randomly selected from the normal and infected libraries for DNA template preparation, respectively. After template quality screening, a total of 6,964 and 7,686 cDNA clones were sequenced from the 3' end from the normal and infected libraries, respectively. After base-calling, vector sequence trimming and screening to eliminate low quality sequences and contamination from WSSV and other sources, a total of 6,658 and 7,276 high quality 3' ESTs were generated from the normal and infected libraries, respectively (Table 1).

After finishing the 3' end sequencing, as well as 3' end sequence assembly and annotation, we next performed DNA sequencing from the 5' end for a fraction of cDNA clones. We randomly chose the cDNA clones from the 3' EST contigs that showed no significant hits of the BlastX searches to the NCBI nr database. From the normal and infected libraries, 1,036 and 1,119 clones, respectively, were subjected to 5' end sequencing with SP6 primer. After base-calling, trimming the vector sequence and eliminating low quality sequences, 978 and 1,069 high quality 5' ESTs were generated (Table 1). These were then combined with the high quality 3' ESTs for the final assembly and annotation.

Overall, these high quality ESTs were derived from 6,671 normal and 7,298 infected cDNA clones. The average lengths of the high quality sequences were 678 bp and 652 bp in the normal and infected libraries, respectively. The CAP3 assembly program produced 9,622 unique sequences. Of these unique sequences, 8,258 were singlets, consisting of only one EST, and the other 1,364 were contigs, consisting of at least two ESTs. Most contigs contained 2–4 ESTs, and the largest contig was formed by 854 ESTs. The average length of the unique sequences was 678 bp (Table 1).

We also noted that when both libraries were checked for contamination by WSSV sequence, 167 ESTs were found to be WSSV contaminants in the PmTwI library (*E *value < 10^-25^, score > 100), whereas there were no WSSV contaminants in PmTwN. This result is consistent with the PCR screening results for the original, unchallenged postlarvae, and it reconfirmed that the shrimp used in the present study were WSSV-free.

### Sequence similarity

BlastX found 2,027 (21.07%) unique sequences similar to known protein sequences in the NCBI nr protein database and 2,026 (21.06%) unique sequences that had GO annotations in the UniProt database. Working backwards from the unique sequences to the original ESTs in the normal library (PmTwN), this translates to 3,022 (45.30%) ESTs that match known protein sequences in the nr database, and 2,870 (43.02%) ESTs with matches in the UniProt database. In the infected library (PmTwI), the corresponding number of matches are 3,338 (45.74%) and 3,202 (43.88%), respectively. These data are summarized in Table 1.

### Identification of the most abundant genes in each library

Table 2 and 3 list the 50 most abundant genes in each library. In the normal library (Table 2), most of these abundant genes can be classified into four major groups, including proteins involved in ATP metabolism, proteins involved in translation, proteins highly or specifically expressed in muscle, and proteins highly or specifically expressed in the HP. These results probably reflect the fact that the shrimp postlarvae were in an active growth stage. During active growth, both energy and cellular translation machinery would be needed to synthesize proteins and build muscle, while the HP would be actively engaged in synthesizing digestive enzymes, hemocyanin and other proteins. By contrast, in the infected library (Table 3), the most abundant genes no longer included the HP proteins, but instead included four other groups: immune-related proteins with chitin-binding or lectin domains, proteins involved in glycolysis, cuticle-related proteins and several different actin genes.

### Identification of genes with differential abundance

Among the known genes represented by EST matches, 360 genes were found exclusively in the normal library, 361 genes were found only in the infected library, and 264 genes were cross expressed in both libraries. Based on the number of homologous ESTs, Fisher's exact test found a significant increase in abundance for 23 genes (Table 4), and a significant decrease for 25 genes (Table 5).

Among the genes with increased abundance, several major groups can be identified, including four proteins with a chitin binding Peritrophin-A domain, seven cuticle-related proteins, four proteins involved in oxidative phosphorylation, two glycolytic enzymes and two ribosomal proteins. Other increased-abundance genes include thioredoxin-1, actin and a protein homologous to CG6055-PA. The decreased-abundance genes can also be classified into several groups, including two SCP calcium-binding proteins, five cytoskeleton/motility-related proteins, three proteins involved in oxidative phosphorylation, three ribosomal proteins and seven proteins that are produced mainly by the HP. These HP-produced proteins include four digestive enzymes, hemocyanin and two immune-related proteins, PmAV and ferritin. Other decreased-abundance genes include opsin and cAMP responsive element binding protein-like 2.

### Functional classification based on Gene Ontology

The putative functions assigned to the unique sequences by the Gene Ontology (GO) classification scheme suggested that in the normal and infected libraries, respectively, 3,397 and 3,571 ESTs map to biological processes, 3,408 and 3,188 ESTs map to cellular components, and 2,012 and 2,375 ESTs map to molecular functions (Table 6). In both libraries, most of the corresponding biological process genes are involved in electron transport, transport, protein metabolism, phosphate metabolism and carbohydrate metabolism (Table 6). Table 6 also shows that most of the cellular component genes encode proteins located in the mitochondria, membrane, ribosome, cytoskeleton, and nucleus, and that most of the molecular function genes are associated with catalytic activity, transporter activity, structural molecules, and metal ion binding.

Analysis of GO categories showed a significant statistical difference (Fisher's exact test; *P *< 0.05) between the normal and infected libraries for several biological processes, including carbohydrate metabolism, signal transduction, response to external stimulus, microtubule-based movement, phosphate metabolism, transport and protein metabolism (Table 6). Among the processes that appear to be increased after WSSV infection, carbohydrate metabolism showed the most significant change, mostly because this GO category includes the proteins involved in the glycolytic pathway as well as proteins with the chitin binding Peritrophin-A domain, and the abundance of both of these groups was highly increased (Table 3 and 4). Two categories, signal transduction and response to external stimulus, showed significantly decreased abundance in the infected library. We note that opsin is included in both of these two categories.

Two molecular functions, structural molecular activity and carbohydrate binding, appear to be elevated significantly in the infected library (Table 6). The structural molecular activity category consisted of cuticle-related proteins (including the highly abundant BCS-1 in the infected library), ribosomal proteins, and cytoskeletal proteins. The carbohydrate-binding category included proteins with C-type lectin (CTL) and CTL-like domains and proteins with the chitin binding Peritrophin-A domain, both of which showed significantly increased abundance in the infected library (Table 3 and 4). Significantly decreased categories included metal ion binding, motor activity, transporter activity, signal transducer activity and protein binding. The metal ion binding category consisted of a diverse array of proteins, including cytochrome c oxidase subunit II, various enzymes, the calcium-binding proteins and cytoskeletal/muscle-related proteins.

In the cellular component group, only the cytoskeleton category was significantly different (Table 6). This category included the cytoskeleton/motility-related proteins, which had a decreased abundance in the infected library.

We have constructed a database to host all the sequence data and the analysis results obtained from this study. The database can be accessed through a web interface [18].

## Discussion

Shrimps are economically important cultured aquatic animals. However, compared to other aquacultured animals, there have been relatively few studies on shrimp genomics. In the present study, a global analysis of 15,981 high-quality shrimp ESTs revealed 2,027 known genes and 7,595 unknown unique sequences. These ESTs will not only be a valuable addition to the current archived sequences from shrimps, but will also provide a major resource for the comparative analysis of gene expression profiles between normal and WSSV-infected shrimps.

To the extend that changes in EST abundance (Table 4 and 5) are predictive of changes in gene expression, then the present data suggest that in *P. monodon *postlarvae, WSSV infection modulates the expression of various kinds of genes. The predicted up-regulated genes include several proteins involved in oxidative phosphorylation, cuticular proteins, a protein with C-type lectin (CTL) and CTL-like domains, proteins with the chitin binding Peritrophin-A domain, two glycolytic enzymes, and thioredoxin-1. The predicted down-regulated genes include several proteins that are synthesized in the HP (digestive enzymes, two immune-related proteins, and the hemocyanin), five cytoskeleton/motility-related proteins, four proteins involved in oxidative phosphorylation, and opsin. Several ribosomal protein genes and actin genes are also predicted to be differentially modulated by WSSV.

### Cuticular proteins

This is the first study to suggest that WSSV infection strongly up-regulates the expression of cuticular proteins. One prominent feature of arthropods is the cuticular covering of the whole body. Cuticles are highly organized structures made of chitin filaments embedded in a proteinaceous matrix, and they are produced as a layered, extracellular secretion from the underlying epidermis [19]. Pathological studies reveal that the cuticular epidermis is one of the main target tissues of WSSV [20]. At the late stage of infection, this tissue is heavily infected, it loses its cellular architecture and becomes necrotic. Loosening of the cuticle and the appearance of white spots in the cuticular epidermis are two of the pathological characters caused by WSSV infection.

The white spots in the cuticle of WSSV-infected shrimp represent abnormal deposits of calcium salts by the cuticular epidermis [21]. Table 4 suggests that after WSSV infection, a gene corresponding to crayfish calcification-associated peptide-1 (CAP-1) is very strongly up-regulated. Crayfish CAP-1 is isolated from the exoskeleton, has chitin-binding ability and, most importantly, anti-calcification activity [22]. CAP-1 mRNA is strongly expressed in the epidermal tissue during the postmolt stage [23]. Inoue et al. [23] proposed that CAP-1 might play an important role in calcification and cuticle formation in the exoskeleton, and if so, then, the abnormal production of CAP-1 in WSSV-infected shrimp may cause the abnormal deposits of calcium salt, leading to the formation of white spots in the cuticle.

Table 4 includes a protein gene that is a homolog to BCS-1, a gene that was cloned from a subtracted barnacle cypris larval cDNA library by differential screening [24]. BCS-1 mRNA is specifically expressed in barnacle cypris larvae, and during the process of larval attachment and metamorphosis, the amount of BCS-1 mRNA is decreased [24]. The function of BCS-1 remains unknown. However, ScanProsite analysis of the BCS-1 protein sequence revealed that BCS-1 contains the chitin-binding type R&R domain profile, which is a structural feature of insect cuticular proteins. This suggests that BCS-1 is a barnacle cuticular protein, and the homolog to BCS-1 in Table 4 is therefore described as a cuticle protein.

Pathological studies have shown that WSSV infection seriously damages the cuticular epidermis, and this study now suggests that WSSV infection strongly up-regulates the gene expressions of various cuticular proteins. It remains unclear how and why WSSV infection induces the expression of cuticular protein genes, and whether this benefits WSSV. These are questions that deserve further study.

### Gene expression in non-primary WSSV-target organs: HP, muscle and compound eye

Compared to the cuticular epidermis, the HP, muscle and compound eye are only lightly infected by WSSV, and these organs remain intact at the late stage of infection [20]. In HP, WSSV mainly infects the myoepithelial cells of the hepatopancreatic sheath and the fibroblast of the connective tissue, whereas the epithelium of the tubules, which synthesize the hemocyanin and digestive enzymes [25], are rarely infected. However, our EST analysis suggests that the RNAs of hemocyanin and several digestive enzymes are strongly reduced after WSSV infection. This suggests that although the epithelium of the tubules are refractory to WSSV infection, at least some of their physiological functions (such as gene transcription) are dramatically affected by the infection of other cell types in HP. If expression of digestive enzymes is down-regulated in HP, then this, together with the fact that WSSV infection targets the stomach, might well explain why WSSV-infected shrimp reduce their food consumption.

The compound eye and muscle are also only lightly infected by WSSV. Even so, it seems that infection severely decreases the transcription of several genes that are primarily and/or highly expressed in these two organs, suggesting that WSSV infection would definitely affect the functions of both organs.

### Proteins with the chitin binding peritrophin-A domain

The chitin binding Peritrophin-A domain is found in chitin binding proteins, particularly the peritrophic matrix proteins of insects and animal chitinases [26,27]. The peritrophic matrix (PM) lines the midgut of insects and it is believed that the PM facilitates digestion and forms a protective barrier to prevent invasion by bacteria, viruses and parasites [28]. There are several classes of PM proteins, and one of these protein classes, the peritrophins, is studied extensively and has been found in several insects [26,27,29]. Recent studies have shown that peritrophin proteins also exist in crustaceans [30-32]. Khayat et al. [30] were the first to identify two peritrophin-like cDNAs that are highly expressed during oogenesis in *Penaeus semisulcatus *and the two proteins are components of the cortical rods, forming a jelly layer after fertilization. A similiar protein was also isolated from the mature ovary of *Marsupenaeus japonicus *[31] Another peritrophin-like protein has also been identified in *Fenneropenaeus chinensis*. This peritrophin mRNA is constitutively expressed in the ovaries, and can only be induced by *E. coli *to express in hemocytes, heart, stomach, gut, and gills [32]. In addition, the recombinant protein can bind to Gram-negative bacteria and chitin, suggesting that it may play a role in immune defense and other physiological responses. Interestingly, as shown in Table 4, the four proteins with a chitin binding Peritrophin-A domain can only be identified in the infected library, which suggests that their expressions are induced after WSSV infection and that they may therefore play some roles in the shrimp's antiviral immune response.

### C-type lectins

In *P. monodon *postlarvae, the protein homologous to Drosophila CG6055-PA showed greatly increased abundance after WSSV infection (Table 4). The Drosophila CG6055-PA protein has a C-type lectin (CTL) and CTL-like domain, which is a structural module that has Ca^2+^-dependent carbohydrate-binding activity. Proteins that have this module are generally known as C-type (Ca^2+^-dependent) lectins to distinguish them from the other (Ca^2+^-independent) types of animal lectins. Animal C-type lectins play important roles in innate and adaptive immunity through pathogen recognition and cellular interactions [33]. In invertebrates, C-type lectins are involved in various immune responses, including the activation of the proPO system [34], antibacterial activity [35], and the promotion of phagocytosis [36]. In shrimps, several C-type lectins have been identified. PmLec is a *P. monodon *C-type lectin that is able to bind to bacterial lipopolysaccharide (LPS) to enhance hemocyte phagocytosis [36]. Fclectin is a C-type lectin gene cloned from the hemocytes of Chinese shrimp *Fenneropenaeus chinensis *[37]. Fclectin mRNA is mainly expressed in hemocytes and its expression is greatly affected after challenge by bacteria, LPS or WSSV [37]. Neither PmLec nor Fclectin was found in our ESTs, but another C-type lectin, PmAV, was represented. The PmAV gene has been identified in WSSV-resistant *P. monodon*, and the recombinant protein shows a strong antiviral activity toward a fish virus *in vitro *[38]. Unlike the CG-6055-PA homolog, however, Table 5 suggests that PmAV was strongly down-regulated. A third C-type lectin, the gene homologous to Drosophila RH18728p, was also identified in our EST database, but its expression does not appear to change significantly after WSSV infection.

### Calcium-binding proteins

Table 5 suggests that expression of the sarcoplasmic calcium-binding protein (SCP) α and β subunits is strongly down-regulated after WSSV infection. Shrimp, lobster and crayfish SCPs exist as dimers of two different polypeptide chains, the α and β [39-41], and they function as cytosolic Ca^2+ ^buffers. In crayfish, although the SCP is ubiquitously expressed in many tissues, it is most abundant in muscle [42]. If the *P. monodon *SCP α and β subunits are also both highly expressed in muscle, then this would be consistent with our observation that, just like several other muscle-specific transcripts, these two genes showed decreased abundance after WSSV infection (Table 5). In this connection, we also note that WSSV itself encodes several proteins with EF-hand calcium-binding motifs [4]. This means that WSSV can potentially modulate the calcium ion concentration of infected cells by decreasing the expression of shrimp cell SCP and by simultaneously expressing the viral-encoded calcium-binding proteins.

### Glycolytic pathway

Until recently, glycolytic enzymes were considered as "straightforward" enzymes with no sophisticated regulatory properties. However, these enzymes are now known to perform various functions in addition to their innate glycolytic function, and they play an important role in several biological and pathophysiological processes [43-45]. Two glycolytic enzyme genes, phosphopyruvate hydratase and fructose 1,6-bisphosphate aldolase, showed increased abundance after WSSV infection. Phosphopyruvate hydratase is a key protein in the glycolytic pathway, catalyzing the conversion of 2-phosphoglycerate to phosphoenopyruvate, but it can also be a receptor for plasminogen [46,47] or a transcriptional repressor [45]. Together with phosphoglycerate kinase and tubulin, phosphopyruvate hydratase forms an active transcription initiation complex that enhances transcriptional elongation of the Sendai virus genome [48]. In addition, this enzyme has also been described as a stress protein induced by hypoxia [49]. The non-glycolytic functions of the other glycolytic enzyme, aldolase, presently remain unknown. Nevertheless, taken together, all these studies suggest that it would be worthwhile to further investigate whether either or both of these enzymes play an essential role during WSSV infection.

### Oxidative phosphorylation

Several proteins involved in oxidative phosphorylation and encoded by mitochondrial DNA showed differential abundance at the RNA level after WSSV infection (Table 4 and 5). WSSV infection decreases the abundance of NADH dehydrogenase subunits 1 and 5 and cytochrome c oxidase subunit II, but simultaneously increased the abundance of cytochrome b, cytochrome c oxidase subunit I and IV and ATPase subunit C. Numerous studies have shown that viruses affect mitochondria in different ways. Morphological changes in mitochondria are induced by human immunodeficiency virus (HIV) [50], human T-cell leukemia virus type 1 [51] and Rubella virus [52]. Changes in location are observed after infection by Human herpesvirus 1 (HHV-1) and Hepatitis B virus [53,54]. The mitochondrial respiratory chain is affected by simian virus 40 [55], Poliovirus [56], HHV-1 and influenza virus [57]. The present study now suggests for the first time that virus infection might also modulate the amounts of mitochondrial mRNAs. However, it remains to be determined whether such changes could affect the mitochondrial oxidative phosphorylation and hence the generation of ATP.

### Actin genes

WSSV infection also differentially affected the abundance of actin genes (Table 4 and 5). Actins are conserved proteins that participate in muscle contraction, cell motility, cell division, and cytoskeletal structure [58]. In almost all eukaryotes, actins are encoded by members of multigene families, and these different actin genes are expressed differently across different cell types, tissues, and developmental stages [59]. In vertebrates, three main groups of actin isoforms, α, β and γ, have been identified. The α-actins are found in muscle tissues and the β and γ-actins coexist in most cell types as components of the cytoskeleton. The actin and the microtubule cytoskeleton play important roles in the life cycle of every virus, from the beginning of virus attachment to the host cell to the final assembly and egress of virus [60]. In spite of this, however, some viruses actively degrade some specific host mRNAs, including β-actin, to shut off host cell protein synthesis [61,62]. Two recent studies have revealed the interplay between WSSV and the shrimp actin gene. One of the major WSSV structural proteins, VP26, interacts with actin [63], and the actin mRNA becomes unstable after WSSV infection [10]. The present study now suggests that the expression of these *P. monodon *actins is differentially modulated after WSSV infection. Two isoforms (gi|113216 and gi|3907620) showed increased abundance (Table 4), whereas one (gi|3907622) showed decreased abundance (Table 5). We also note that relatively speaking, the *P. monodon *actins are not very well documented. For instance, while *Artemia *is shown to have 8–10 actin genes and 4 isoforms, one of which is muscle specific, and crab (*Gecarcinus latefalis*) has seven or eight documented actin genes [64], only two *P. monodon *actin genes have so far been released to a public database. The previously undocumented actin genes listed in our EST libraries (see Table 2) now add several more actins to that list.

The predicted modulation of actin isoforms by WSSV also means that actin is not a good choice of reference gene in RNA level studies of WSSV virus/host interactions. This is in addition to the already known difficulty that while the vertebrate muscular actins are recognizable from their amino acid sequence, the invertebrate actins cannot be distinguished in the same way because they all resemble the vertebrate cytoplasmic actins [65,66]. Clearly, further studies will be needed to characterize and classify these *P. monodon *actin genes by their tissue distribution patterns, with a particular focus on the isoforms that have their expressions modulated by WSSV.

## Conclusion

In conclusion, the 15,981 high-quality ESTs generated in this study provide a rich source for identification of novel genes in shrimp and for comparative analysis of gene expression patterns in normal and WSSV-infected shrimp. An EST-based strategy not only greatly facilitates *in silico *expression profiling, it also provides an experimental approach to elucidate WSSV pathogenesis and to investigate the shrimp's response to virus infection. Our data suggest that in postlarval shrimp, WSSV infection strongly affects the physiological functions of several organs/tissues, including the HP, muscle, eyestalk and cuticle, and that the expressions of several genes in these organs/tissues are strongly modulated. In addition, WSSV is predicted to affect several basic cellular metabolic processes, including oxidative phosphorylation, protein synthesis, glycolysis, and calcium ion balance.

## Methods

### Shrimp, virus and challenge

The postlarvae used to construct the cDNA libraries were at the PL20 stage, with an average length and weight of 9 mm and 0.02 g, respectively. These postlarvae were derived from WSSV-free *Penaeus monodon *spawners. They were cultivated in the Tungkang marine laboratory of Taiwan's Fisheries Research Institute. The WSSV suspension used to challenge the postlarvae was prepared from frozen (-80°C), WSSV-infected black tiger shrimp that had been collected during a natural outbreak of WSS in 1994 [67]. The postlarvae were challenged by immersion. At 66 hours after infection, they were collected in cryotubes and stored in liquid nitrogen for later RNA extraction. They were further confirmed by PCR to be WSSV-infected. Normal, WSSV-free postlarvae were also collected and stored in liquid nitrogen and their WSSV-free status was confirmed by PCR.

### RNA extraction and cDNA library construction

For each library, RNA was extracted from a pool of about 50 entire animals weighing approximately 1 g in total. Total RNAs were extracted from normal and WSSV-infected postlarvae using RNAzol B reagent (Teltest Ltd. Friendswood, TX), and the mRNAs were purified from the total RNAs using the QuickPrep™ Micro mRNA Purification Kit (GE healthcare) following the protocol provided by the manufacturer. The respective cDNA libraries were constructed using the λZAP-cDNA library construction kit (Stratagene) according to the manufacturer's instructions. In brief, the first-strand cDNA was synthesized from 5 μg of mRNA with the oligo-d(T) linker-primer, 5'-(GA)_10_ACTAGTCTCGAG(T)_18_-3', and the MMLV (Molony murine leukemia virus) reverse transcriptase supplied in the kit. The second strand of cDNA was synthesized with DNA polymerase I in the presence of RNase H, and then the cDNA was blunt ended with pfu DNA polymerase. Both strands of the cDNA were ligated with the *Eco*R I adapter, and digested by *Xho *I. cDNA fractionation was performed using Sepharose CL-4B gel filtration, and the cDNA was then inserted into the *Eco*R I-*Xho *I site of the Uni-Zap phage vector. The resulting phage libraries were converted to pBluescript phagemid libraries by massive *in vivo *excision using ExAssist helper phage. About 8,000 white colonies were randomly isolated from both normal and infected libraries on LB-ampicillin plates containing IPTG and X-gal, and plasmid DNA was extracted from each library.

### Sequencing

Randomly selected clones were inoculated into individual wells of 96-well plates with 170 μl LB media containing 8 μg ampicillin, and the plates were incubated at 37°C for 18 hours. The DNA templates were prepared using Montage Plasmid Miniprep_96 _kits, and checked by electrophoresis in 1% agarose gel. Sequencing was carried out using the BigDye version 3.0 sequencing reaction kit using the optimal protocol provided by the manufacturer. DNA sequencing from the 3' end and 5' end of the cDNA was conducted with T7 or SP6 primers, respectively, on a high-throughput automated sequencer (MJ Research BaseStation and ABI3730, USA) using standard protocols.

### Sequence analysis

The raw traces for ESTs were subjected to base-calling by running Phred (Q > 13). pBluescript vector sequences were trimmed using Cross_match with default parameters (minimatch 12, penalty -2, minscore 20). Those ESTs having a length of more than 100 bp after vector trimming were subject to further analysis. BlastN [68] was used to find matching sequences with *E *value < 10^-5 ^in the whole WSSV genome (GenBank accession no. AF440570), and also to screen out possible contaminants from bacterial chromosomal DNA, RNA, and lambda phage DNA. The interspersed repeats and low complexity sequences in the ESTs were then masked by RepeatMasker using the Drosophila repeat sequence as reference. Low quality sequences, including short sequences (less than 100 bp) and those with a high percentage of nucleotide A (adenosine) or N (uncertain read) were considered uninformative and were eliminated from further analysis. The ESTs that passed through the above quality check procedures were considered high quality ESTs. The high quality ESTs in both the normal and infected libraries were combined and assembled to form contigs using CAP3 [69] with the overlapping percentage parameter set to > 95% in order to obtain highly reliable contig sequences. ESTs that did not form contigs designated singlets. Collectively, the resultant contigs and singlets are referred to as unique sequences.

### Functional annotation

Putative functions of the unique sequences were discovered by using BlastX to translate each nucleotide query sequence into all reading frames and then searching for matches in the NCBI non-redundant database. Significant hits (with *E *value < 10^-10^) in the NCBI nr database were followed up with protein function searches in the UniProt database [70], which provides value-added information reports for protein functions. The UniProt reports consist of Gene Ontology (GO) annotations that classify proteins by biological process, cellular component, and molecular function. Each unique sequence was tentatively assigned GO classification based on annotation of the single "best hit" match in UniProt. These data were then used to classify the corresponding genes according to their GO functions.

### Expression analysis and statistical evaluation of EST occurrence

The unique sequences were considered to have increased abundance if they had a significantly greater number of hits (ie. more ESTs) in the WSSV-infected library compared to the normal library. Conversely, unique sequences were considered to have decreased abundance if they had significantly more hits in the normal library. The statistical significance of homologous ESTs with differential abundance was determined using Fisher's exact test [71-73], which is widely used to evaluate 2 × 2 contingency tables. Fisher's exact test produces a significance value *P *ranging between 0 and 1, where a value close to 0 implies that there is a significant differential abundance of the gene or the annotated function between the normal and infected libraries. The significance of differential abundance genes with a *P *value smaller than 0.001 was considered "very strong". *P *values between 0.001 and 0.01, and between 0.01 and 0.05 were considered "strong" and "moderate", respectively.

## Availability and requirements

Project name: *Penaeus monodon *Functional Genomics Database

Project home page: 

Operating system: Platform independent

Programming language:PHP

## Abbreviations

WSSV: white spot syndrome virus

HP: hepatopancreas

## Authors' contributions

JHL extracted RNAs from shrimps, and constructed the cDNA library. IH and TA carried out the sequence analysis. CCC, CWH and HCH carried out the sequence analysis, functional annotation, and expression analysis. CFL and GHK conceived and directed the project. CFL and HCH designed the study. JHL, JLW, CCC, HFJ, and HCH drafted the manuscript. All authors read and approved the final manuscript.

## Figures and Tables

**Table 1:** Summary of cDNA libraries and EST data

	Library		
	
	PmTwN		PmTwI
	
Category	Number of sequences		
Total sequenced cDNA^1^	3':6964, 5':1036		3':7686, 5':1119
High quality ESTs^1^	3':6658, 5': 978		3':7276, 5':1069
cDNA clones that produced high quality ESTs	6671		7298
Mean EST length (bp)	678		652
Unique sequences		9622	
Mean unique sequence length (bp)		678	
Contigs		1364	
Matched unique sequence (nr db)		2027 (21.07%^2^)	
Matched unique sequence (UniProt db)		2026 (21.06%^2^)	
Matched ESTs^3 ^(nr db)	3022 (45.30%^4^)		3338 (45.74%^4^)
Matched ESTs^3 ^(UniProt db)	2870 (43.02%^4^)		3202 (43.88%^4^)

^1^: The numbers of 3' and 5' end sequences are shown.

^2^: The percentage is calculated by dividing the number of unique sequences (9622) by the number of matched unique sequences (2027 and 2026, respectively).

^3^: Some cDNA clones have both 5' and 3' ESTs, and when both or either ESTs had matches in databases, the corresponding 5' and 3' ESTs from the same clone will be marked and only one was counted.

^4^: The percentage is calculated by dividing the number of cDNA clones that produced high quality ESTs (6671 or 7298) by the number of matched ESTs (3022 and 3338, respectively, or 2870 and 3202).

**Table 2:** The fifty most abundant genes in the normal library.

**Gene Name**	**Species**	**Acc. No.**	**putative feature/function**	**EST no.**
cytochromec oxidase subunit I	*Penaeus monodon*	gi|7374116	ATP metabolism	366
cytochromec oxidase subunit II	*Penaeus monodon*	gi|7374117	ATP metabolism	305
cytochromec oxidase subunit III	*Penaeus monodon*	gi|7374118	ATP metabolism	164
ATP synthase F0 subunit6	*Penaeus monodon*	gi|7374114	ATP metabolism	89
NADH dehydrogenase subunit5	*Penaeus monodon*	gi|7374125	ATP metabolism	83
NADH dehydrogenase subunit 1	*Penaeus monodon*	gi|7374120	ATP metabolism	81
NADH dehydrogenase subunit 4	*Penaeus monodon*	gi|7374123	ATP metabolism	70
cytochrome b	*Penaeus monodon*	gi|7374119	ATP metabolism	54
NADH dehydrogenase subunit 2	*Penaeus monodon*	gi|7374121	ATP metabolism	22
ADP-ATP translocator	*Ethmostigmus rubripes*	gi|15559050	ATP metabolism	14
NADH dehydrogenase subunit 6	*Penaeus monodon*	gi|7374126	ATP metabolism	12
cytochrome oxidase subunit I	*Fenneropenaeus sp. HCCP-2002*	gi|21666426	ATP metabolism	9
actin 2	*Penaeus monodon*	gi|3907622	cytoskeleton/motility	41
Chain B, Apocrustacyanin C1 subunit	*Homarus gammarus*	gi|33357653	cytosolic fatty-acid binding protein	10
cAMP responsive element binding protein-like 2	*Homo sapiens*	gi|4503035	DNA binding/tumor suppressor	13
opsin	*Procambarus clarkii*	gi|263970	eyestalk/vision	16
phosphopyruvate hydratase	*Penaeus monodon*	gi|3885968	glycolysis	13
zinc proteinase Mpc1	*Paralithodes camtschaticus*	gi|19774211	HP/digestive enyyme	11
trypsin	*Litopenaeus vannamei*	gi|3006086	HP/digestive enzyme	19
trypsin	*Litopenaeus vannamei*	gi|3006084	HP/digestive enzyme	16
Chymotrypsin BII	*Litopenaeus vannamei*	gi|2462649	HP/digestive enzyme	7
Ferritin	*Pacifastacus leniusculus*	gi|26006755	HP/immune-related	10
PMAV	*Penaeus monodon*	gi|34576191	HP/immune-related	7
hemocyanin	*Penaeus monodon*	gi|16612121	HP/oxygen transfer	62
hemocyanin	*Litopenaeus vanname*	gi|854403	HP/oxygen transfer	34
hemocyanin	*Litopenaeus vannamei*	gi|7414468	HP/oxygen transfer	20
LIM protein	*Apriona germari*	gi|50982101	muscle	15
SCP, beta chain	*Penaeus sp.*	gi|134315	muscle/calcium-binding	49
SCP, alpha-B and -A chains	*Penaeus sp*.	gi|134312	muscle/calcium-binding	40
MLC1 protein	*Anopheles gambiae*	gi|19572388	muslce	35
Myosin light chain 2	*Drosophila melanogaster*	gi|16648286	muscle/cytoskeleton/motility	51
Troponin I	*Pontastacus leptodactylus*	gi|136223	muscle/cytoskeleton/motility	31
Troponin C, isotype gamma	*Pontastacus leptodactylus*	gi|136032	muscle/cytoskeleton/motility	19
slow muscle myosin S1 heavy chain	*Homarus americanus*	gi|37925239	muscle/cytoskeleton/motility	16
fast myosin heavy chain	*Homarus americanus*	gi|414985	muscle/cytoskeleton/motility	15
fast tropomyosin isoeorm	*Homarus americanus*	gi|2660868	muscle/cytoskeleton/motility	8
arginine kinase	*Penaeus monodon*	gi|27463265	muscle/energy pathway	28
Translation elongation factor EF-1alpha	*Danio rerio*	gi|38174284	translation	18
nascent-polypeptide-associated complex alpha polypeptide	*Homo sapiens*	gi|5031931	translation	10
ribosomal protein eL12	*Artemia sp*.	gi|5689	translation	13
ribosomal protein L37	*Spodoptera frugiperda*	gi|15213792	translation	12
Ribosomal_L22	*Drosophila yakuba*	gi|38047573	translation	12
Ribosomal L14	*Danio rerio*	gi|29294663	translation	11
ribosomal protein L23	*Homo sapiens*	gi|13097600	translation	11
ribosomal protein S21	*Branchiostoma belcheri tsingtaunese*	gi|30267907	translation	10
Rps16 protein	*Mus musculus*	gi|52078405	translation	9
RpL9	*Drosophila yakuba*	gi|38047669	translation	8
Ribosomal protein S17	*Homo sapiens*	gi|38541200	translation	8
Ribosomal protein S24	*Danio rerio*	gi|51980430	translation	7
ribosomal protein L35A	*Spodoptera frugiperda*	gi|15213788	translation	^7^

**Table 3:** The fifty most abundant genes in the WSSV-infected library.

**GeneName**	**Species**	**Acc. No.**	**putative feature/function**	**EST no.**
cytochromec oxidase subunit I	*Penaeus monodon*	gi|7374116	ATP metabolism	488
cytochromec oxidase subunit II	*Penaeus monodon*	gi|7374117	ATP metabolism	270
cytochromec oxidase subunit III	*Penaeus monodon*	gi|7374118	ATP metabolism	174
cytochrome b	*Penaeus monodon*	gi|7374119	ATP metabolism	112
ATP synthase F0 subunit 6	*Penaeus monodon*	gi|7374114	ATP metabolism	86
NADH dehydrogenase subunit4	*Penaeus monodon*	gi|7374123	ATP metabolism	60
NADH dehydrogenase subunit 5	*Penaeus monodon*	gi|7374125	ATP metabolism	56
NADH dehydrogenase subunit 1	*Penaeus monodon*	gi|7374120	ATP metabolism	43
NADH dehydrogenase subunit 2	*Penaeus monodon*	gi|7374121	ATP metabolism	13
cytochrome oxidase subunit I	*Fenneropenaeus sp*	gi|21666426	ATP metabolism	13
ATP lipid-binding protein like protein	*Marsupenaeus japonicus*	gi|18700491	ATP metabolism	10
NADH dehydrogenase subunit 6	*Penaeus monodon*	gi|7374126	ATP metabolism	9
Cytochrome c oxidase subunit IV	*Drosophila melanogaster*	gi|16197913	ATP metabolism	8
ADP-ATP translocator	*Ethmostigmus rubripes*	gi|15559050	ATP metabolism	7
CG14607-PA	*Drosophila melanogaster*	gi|24644788	Chitin binding Peritrophin-A domain/immune	13
ENSANGP00000013986	*Anopheles gambiae*	gi|31243037	Chitin binding Peritrophin-A domain/immune	10
ENSANGP00000023091	*Anopheles gambiae*	gi|31200471	Chitin binding Peritrophin-A domain/immune	8
CG6055-PA	*Drosophila melanogaster*	gi|7297257	C-type lectin (CTL) and CTL-like domains/immune	18
BCS-1	*Balanus amphitrite*	gi|9186884	cuticle protein	324
Endocuticle structural glycoprotein SgAbd-8	*Schistocerca gregaria*	gi|47605412	cuticle protein	32
calcification-associated peptide-1	*Procambarus clarkii*	gi|33468738	cuticle protein	17
Cuticle protein AMP1A	*Homarus americanus*	gi|3287772	cuticle protein	11
DD9B	*Marsupenaeus japonicus*	gi|7008007	cuticle protein	10
cuticle protein 20	*Manduca sexta*	gi|19548965	cuticle protein	9
actin1	*Penaeus monodon*	gi|3907620	cytoskeleton/motility	17
Actin	*Bombyx mori*	gi|113216	cytoskeleton/motility	14
beta actin	*Homarus gammarus*	gi|34576243	cytoskeleton/motility	9
fructose 1,6-bisphosphate aldolase	*Homalodisca coagulata*	gi|46561746	glycolysis	69
phosphopyruvate hydratase	*Penaeus monodon*	gi|3885968	glycolysis	46
glyceraldehyde-3-phosphate dehydrogenase	*Procambarus clarkii*	gi|31338868	glycolysis	10
triosephosphate isomerase	*Archaeopotamobius sibirien*	gi|19848023	glycolysis	9
trypsin	*Litopenaeus vannamei*	gi|3006086	HP/digestive enyyme	9
LIM protein	*Apriona germari*	gi|50982101	muscle	31
SCP, alpha-B and -A chains	*Penaeus sp.*	gi|134312	muscle/calcium-binding	18
MLC1 protein	*Anopheles gambiae*	gi|19572388	muscle/cytoskeleton/motility	34
slow muscle myosin S1 heavy chain	*Homarus americanus*	gi|37925239	muscle/cytoskeleton/motility	11
Troponin I	*Pontastacus leptodactylus*	gi|136223	muscle/cytoskeleton/motility	9
arginine kinase	*Penaeus monodon*	gi|27463265	muscle/enery pathway	40
thioredoxin-1	*Mesobuthus cyprius*	gi|51869033	redox reaction	14
Translation elongation factor EF-1alpha (GTPase)	*Danio rerio*	gi|38174284	translation	14
Ribosomal L18p	*Xenopus laevis*	gi|27371036	translation	13
Ribosomal protein S24	*Danio rerio*	gi|51980430	translation	12
ribosomal protein eL12	*Artemia sp.*	gi|5689	translation	9
RpL9	*Drosophila yakuba*	gi|38047669	translation	9
ribosomal protein L35A	*Spodoptera frugiperda*	gi|15213788	translation	8
40S ribosomal protein S10	*Ictalurus punctatus*	gi|15294031	translation	8
ribosomal protein L7	*Spodoptera frugiperda*	gi|18253049	translation	8
Ribosomal L22	*Drosophila yakuba*	gi|38047573	translation	7
Rps16 protein	*Mus musculus*	gi|52078405	translation	7
porin	*Anopheles gambiae*	gi|19697919	voltage dependent anion-selective channel	7

**Table 4:** Unique genes with increased differential abundance in the normal and infected libraries.

**GeneName**	**Acc. no.**	**putative feature/function**	**No. of EST in normal**	**No. of EST in normal**	***P *value**	Degree*
cytochrome b	gi|7374119	ATP metabolism	54 (0.81%)	112 (1.54%)	< 0.001	V. S.
cytochrome c oxidase subunit I	gi|7374116	ATP metabolism	366 (5.49%)	488 (6.69%)	0.003	S.
Cytochrome c oxidase subunit IV	gi|16197913	ATP metabolism	0 (0.00%)	8 (0.11%)	0.008	S.
ATPase subunit C	gi|18700491	ATP metabolism	1 (0.02%)	10 (0.14%)	0.013	M.
ENSANGP00000013986	gi|31243037	Chitin binding Peritrophin-A domain	0 (0.00%)	10 (0.14%)	0.002	S.
ENSANGP00000023091	gi|31200471	Chitin binding Peritrophin-A domain	0 (0.00%)	8 (0.11%)	0.008	S.
RE51076p	gi|21064537	Chitin binding Peritrophin-A domain	0 (0.00%)	7 (0.10%)	0.016	M.
CG14607-PA	gi|24644788	Chitin binding Peritrophin-A domain	0 (0.00%)	13 (0.18%)	< 0.001	V. S.
CG6055-PA	gi|7297257	C-type lectin (CTL) and CTL-like domain	1 (0.02%)	18 (0.25%)	< 0.001	V. S.
BCS-1	gi|9186884	cuticle protein	4 (0.06%)	324 (4.44%)	< 0.001	V. S.
Endocuticle structural glycoprotein SgAbd-8	gi|47605412	cuticle protein	2 (0.03%)	32 (0.44%)	< 0.001	V. S.
calcification-associated peptide-1	gi|33468738	cuticle protein	0 (0.00%)	17 (0.23%)	< 0.001	V. S.
Cuticle protein AMP1A	gi|3287772	cuticle protein	0 (0.00%)	11 (0.15%)	0.001	S.
DD9B	gi|7008007	cuticle protein	1 (0.02%)	10 (0.14%)	0.013	M.
cuticle protein 20	gi|19548965	cuticle protein	1 (0.02%)	9 (0.12%)	0.023	M.
Cuticle protein AM/CP1114	gi|5921935	cuticle protein	0 (0.00%)	6 (0.08%)	0.032	M.
Actin	gi|113216	cytoskeleton/motility	0 (0.00%)	14 (0.19%)	< 0.001	V. S.
actin 1	gi|3907620	cytoskeleton/motility	5 (0.08%)	17 (0.23%)	0.019	M.
fructose 1,6-bisphosphate aldolase	gi|46561746	glycolysis	5 (0.08%)	69 (0.95%)	< 0.001	V. S.
phosphopyruvate hydratase	gi|3885968	glycolysis	13 (0.20%)	46 (0.63%)	< 0.001	V. S.
thioredoxin-1	gi|51869033	redox reaction	2 (0.03%)	14 (0.19%)	0.005	S.
ribosomal protein L10A	gi|14994666	translation	0 (0.00%)	6 (0.08%)	0.032	M.
ribosomal protein L7	gi|18253049	translation	1 (0.02%)	8 (0.11%)	0.041	M.

*: The degree of significance is based on *P *value. V. S.: very strong (*P *< 0.001), S.: strong (0.001 ≦ < 0.01), M.: moderate (0.01 ≦ *P *< 0.05).

**Table 5:** Unique genes with decreased differential abundance in the normal and infected libraries.

**GeneName**	**Acc. no.**	**putative feature/function**	**No.of EST in normal**	**No.of EST in normal**	***P *value**	Degree*
NADH dehydrogenase subunit 1	gi|7374120	ATP metabolism	81 (1.21%)	43 (0.59%)	< 0.001	V. S.
NADH dehydrogenase subunit 5	gi|7374125	ATP metabolism	83 (1.24%)	56 (0.77%)	0.005	S.
cytochrome c oxidase subunit II	gi|7374117	ATP metabolism	305 (4.57%)	270 (3.70%)	0.010	M.
actin 2	gi|3907622	cytoskeleton	41 (0.62%)	4 (0.06%)	< 0.001	V. S.
cAMP responsive element binding protein-like 2	gi|4503035	DNA binding/tumor suppressor	13 (0.20%)	4 (0.06%)	0.026	M.
opsin	gi|263970	eyestalk/vision	16 (0.24%)	0 (0.00%)	< 0.001	V. S.
trypsin	gi|3006084	HP/digestive enyme	16 (0.24%)	0 (0.00%)	< 0.001	V. S.
zinc proteinase Mpc1	gi|19774211	HP/digestive enyme	11 (0.17%)	0 (0.00%)	< 0.001	V. S.
Chymotrypsin BII	gi|2462649	HP/digestive enyme	7 (0.11%)	0 (0.00%)	0.006	S.
trypsin	gi|3006086	HP/digestive enyme	19 (0.29%)	9 (0.12%)	0.037	M.
PmAV	gi|34576191	HP/immune-related	7 (0.11%)	0 (0.00%)	0.006	S.
Ferritin	gi|26006755	HP/immune-related	10 (0.15%)	3 (0.04%)	0.049	M.
hemocyanin	gi|16612121	HP/oxygen transfer	62 (0.93%)	0 (0.00%)	< 0.001	V. S.
hemocyanin	gi|854403	HP/oxygen transfer	34 (0.51%)	0 (0.00%)	< 0.001	V. S.
hemocyanin	gi|7414468	HP/oxygen transfer	20 (0.30%)	0 (0.00%)	< 0.001	V. S.
SCP, beta chain	gi|134315	muscle/calcium-binding	49 (0.74%)	2 (0.03%)	< 0.001	V. S.
SCP, alpha-B and -A chains	gi|134312	muscle/calcium-binding	40 (0.60%)	18 (0.25%)	0.001	S.
Myosin light chain 2	gi|16648286	muscle/cytoskeleton/motility	51 (0.77%)	3 (0.04%)	< 0.001	V. S.
fast myosin heavy chain	gi|414985	muscle/cytoskeleton/motility	15 (0.23%)	0 (0.00%)	< 0.001	V. S.
Troponin I	gi|136223	muscle/cytoskeleton/motility	31 (0.47%)	9 (0.12%)	< 0.001	V. S.
Troponin C, isotype gamma	gi|136032	muscle/cytoskeleton/motility	19 (0.29%)	4 (0.06%)	0.001	S.
slow-tonic S2 myosin heavy chain	gi|46486938	muscle/cytoskeleton/motility	5 (0.08%)	0 (0.00%)	0.025	M.
Ribosomal protein S17	gi|38541200	translation	8 (0.12%)	1 (0.01%)	0.017	M.
Ribosomal L14	gi|29294663	translation	11 (0.17%)	3 (0.04%)	0.029	M.
ribosomal protein S21	gi|30267907	translation	10 (0.15%)	3 (0.04%)	0.049	M.

**Table 6:** Gene Ontology of the sequences with significant Blastx hits in UniProt

**GO Categories**	**Normal**	**Infected**	***P *value**
**Biological process**			
protein modification	44 (0.66%)	49 (0.67%)	1.00
nucleic acid metabolism	0 (0.00%)	0 (0.00%)	1.00
electron transport	1236 (18.53%)	1348 (18.47%)	0.93
cell adhesion	12 (0.18%)	9 (0.12%)	0.51
nucleosome assembly	6 (0.09%)	10 (0.14%)	0.46
lipid metabolism	13 (0.19%)	21 (0.29%)	0.30
protein metabolism**	506 (7.59%)	490 (6.71%)	0.05
transport**	1125 (16.86%)	1133 (15.52%)	0.03
phosphate metabolism**	247 (3.70%)	219 (3.00%)	0.02
microtubule-based movement**	3 (0.04%)	16 (0.22%)	0.01
response to external stimulus**	39 (0.58%)	14 (0.19%)	0.00
signal transduction**	72 (1.08%)	30 (0.41%)	0.00
carbohydrate metabolism **	94 (1.41%)	232 (3.18%)	0.00
**Cellular component**			
mitochondrion	1347 (20.19%)	1434 (19.65%)	0.43
ribosome	258 (3.87%)	299 (4.10%)	0.52
cytoskeleton**	193 (2.89%)	132 (1.81%)	0.00
nucleus	73 (1.09%)	93 (1.27%)	0.35
membrane	1273 (19.08%)	1384 (18.96%)	0.86
extracellular matrix	3 (0.04%)	1 (0.01%)	0.35
extracellular region	39 (0.58%)	62 (0.85%)	0.07
unlocaliyed protein complex	2 (0.03%)	3 (0.04%)	1.00
**Molecular component**			
peroxidase activity	9 (0.13%)	4 (0.05%)	0.17
carbohydrate binding**	41 (0.61%)	75 (1.03%)	0.01
metal ion binding**	625 (9.37%)	490 (6.71%)	0.00
nucleic acid binding	157 (2.35%)	157 (2.15%)	0.42
nucleotide binding	133 (1.99%)	139 (1.90%)	0.71
protein binding**	97 (1.45%)	77 (1.06%)	0.04
catalytic activity	1716 (25.72%)	1886 (25.84%)	0.88
enyyme regulator activity	14 (0.21%)	12 (0.16%)	0.56
motor activity**	127 (1.90%)	79 (1.08%)	0.00
obsolete molecular function	0 (0.00%)	0 (0.00%)	1.00
antimicrobial peptide activity	0 (0.00%)	0 (0.00%)	1.00
signal transducer activity**	43 (0.64%)	21 (0.29%)	0.00
structural molecule activity**	367 (5.50%)	860 (11.78%)	0.00
transporter activity**	1405 (21.06%)	1358 (18.61%)	0.00
translation regulator activity	56 (0.84%)	45 (0.62%)	0.13

** indicates statistically significant GO term with *P *< 0.05 evaluated by Fisher's exact test
